# Association Between Farming Activities and *Plasmodium falciparum* Transmission in Rural Communities in Nigeria

**DOI:** 10.21315/mjms2020.27.3.11

**Published:** 2020-06-30

**Authors:** Olarewaju Abdulkareem Babamale, Olufunke Adenike Opeyemi, Abiodun Adebayo Bukky, Akinkunmi Idris Musleem, Eniola Olashile Kelani, Blessing Jesuseme Okhian, Nurhidanatasha Abu-Bakar

**Affiliations:** 1 Parasitology Unit, Department of Zoology, University of Ilorin, Ilorin, Kwara State, Nigeria; 2 School of Health Sciences, Universiti Sains Malaysia, Kubang Kerian, Kelantan, Malaysia

**Keywords:** rural, agriculture, malaria parasite, Plasmodium falciparum, transmission

## Abstract

**Background:**

The connection between malaria-associated morbidities and farming activities has not been succinctly reported. This study aimed to address the connectivity between farming activities and malaria transmission.

**Methods:**

The study took place in the agricultural setting of Nigeria Edu local government (9° N, 4.9° E) between March 2016 and December 2018. A pre-tested structured questionnaire was administered to obtain information on their occupation and malaria infection. Infection status was confirmed with blood film and microscopic diagnosis of *Plasmodium falciparum* was based on the presence of ring form or any other blood stages. Individuals who are either critically ill or lived in the community less than 3 months were excluded from the study.

**Results:**

Of the 341 volunteers, 58.1% (52.9% in Shigo and 61.4% in Sista) were infected (parasitaemia density of 1243.7 parasites/μL blood). The prevalence and intensity of infection were higher among farmers (71.3%, 1922.9 parasites/μL blood, *P* = 0.005), particularly among rice farmers (2991.6 parasites/μL blood) compared to non-farmer participants. The occurrence and parasite density follow the same pattern for sex and age (*P* < 0.05). Children in the age of 6 to 10 years (AOR: 2.168, CI: 1.63–2.19) and ≥ 11 years (AOR: 3.750, CI: 2.85–3.80) groups were two-and four-fold more likely to be infected with malaria. The analysis revealed that the proximity of bush and stagnant water to the farmer (73.9%, AOR: 3.242, CI: 2.57–3.61) and non-farmer (38.1%, AOR: 1.362, CI: 1.25–1.41) habitations influence malaria transmission.

**Conclusion:**

This study highlights farming activities as a risk factor for malaria infection in agro-communities. Integrated malaria control measures in agricultural communities should therefore include water and environmental management practices.

## Introduction

Malaria remains as one of the most important parasitic infections in the world with *Plasmodium falciparum* (*P. falciparum*) accounts for the majority of malaria-associated morbidities and mortalities, particularly in holoendemic regions (1). The infection has variable clinical features ranging from a mild febrile illness to life-threatening severe anaemia, acidosis and organ failure among individuals with little or no acquired immunity (2). Total malaria-related deaths stood at 405,000 in 2018 and *P. falciparum* accounted for 99.7% of the estimated malaria cases in the World Health Organization (WHO) of African region, in which 50%, 71% and 65% of cases occurred in South-East Asia, Eastern Mediterranean and Western Pacific, respectively (3, 4).

Of the six countries that accounted for more than half of all malaria cases worldwide as of 2018, Nigeria recorded 25% followed by 12% in the Democratic Republic of the Congo, 5% in Uganda and 4% each in Côte d’Ivoire, Mozambique and Nigeria (5). In Nigeria, a child dies every two minutes, more than millions are indisposed for several days and more than half of all school absenteeism is due to malaria infection (6). The fight against malaria is widely recognised as a pivotal of global development; malaria eradication could save 11 million lives every year and enable re-allocation of billions of US Dollar into other sectors of the economy (6). Therefore, continuous epidemiological surveillance of this deadly disease for as long as infection persists, particularly in high transmission zone is exigent for global exploitation in the evidence-based integration of malaria control approach.

Previous studies have shown that malaria burden is typically common in agricultural areas (7). Farming activities, which take place mostly in the rainy season favour the breeding of mosquitoes and this makes the morbidity of infection apparently noticeable in rural areas due to their proximity to farmlands, particularly in areas where water management practice is pitiable. Thus, farming and malaria transmission are inextricably linked to rainfall, thereby placing farm workers at significant risk of malaria infection (8).

The influence of agricultural activities on the transmission of malaria infection has been documented in many countries of the world (9–11), but it is largely insufficient in the Kwara State, Nigeria. This information is inevitable in the formulation and implementation of the control programme. Therefore, this study aimed at bridging the highlighted gap for the effective design of preventive and control measures in malaria high transmission zones of North Central Nigeria and other endemic countries of the world.

## Methods

### Study Area

The study was conducted in the Bacita community, Edu local government area of Kwara State, located about 79.4 km from Ilorin, the state capital with the geographical coordinates of latitude 9° N, longitude 4.9° E and altitude of 101 m semi-arid land between March 2016 and December 2018. This community in North Central of Nigeria is one of the major agricultural zones of Kwara State. The climate of the study area is typically tropical with rainy (April–September) and dry (October–March) seasons. The majority of the inhabitants are Nupe, Hausa and a few are Yoruba and Igbo. Our study participants are primarily females (62.5%), uneducated (59.5%) with 67.4% peasant farmers growing crops such as rice, beans and sugar cane with little wage (Table 1). The community is inadequately provided with essential amenities such as electricity, good roads and portable water supply. N’dafa River is the major source of water supply for farming and other domestic purposes including drinking. Generally, the sanitation status of the area is poor where bushes are found around houses and bathrooms are mostly sited close to the main building leading to stagnation of water around places of residence.

### Study Design and Sample Collection and Processing

Prior to the study, the community leader and medical personnel in Shigo, Makwagi, Sista and Ogo-Oluwa villages in Bacita community were visited and briefed on the concept and benefit of the study. The participation in this study was made voluntary and the volunteers were randomly screened based on the study criteria. All farmers and non-farmers who live in the rural communities in Nigeria for more than three months were recruited. Individuals that had not stayed in the community for more than three months or critically ill that were unable to respond to the interview were excluded. After the participant screening, 341 participants were considered suitable for represent the entire estimated population of 6,210 people living in our study population with confidence interval and percentage of 5.16 and 48%, respectively.

Blood samples of the consented participants were collected by pricking the thumb with a sterilised lancet for the preparation of thin and thick films on grease-free microscopic slides. Information on the demographic, transmission factors and knowledge about malaria infection were obtained with pre-tested structured questionnaires (Appendix 1). A blood film was considered *P. falciparum*-positive if the ring form or any other blood stages is present by identifying using the criteria previously described (12). The intensity of malaria was recorded by using the standard parasitaemia counting method (13).

### Data Analysis

The prevalence of falciparum malaria was calculated as the proportion of positive samples. The data generated were presented by using descriptive statistics and subjected to Chi-square statistical and one-way analysis of variance (ANOVA) by using Statistical Package for Social Science (SPSS) version 16 to determine the significance in the relationship of infection and intensity rate with age, gender, and other socio-demographic and economic factors. A *P*-value of less than 0.05 was considered significant. A logistic regression analysis (bivariate analysis) was done to obtain the odd ratio for the comparative analysis of risk factors among farmer and non-farmers participants.

## Results

### Prevalence and Parasitaemia Load of P. falciparum Stratified by Location, Occupation and Type of Farming Activities

Blood smears of 341 volunteers were screened for the presence of *P. falciparum* and the results of microscopic examination identified 198 (58.1%) positive cases, which ranged from 52.9% in Shigo to 61.4% in Sista and the overall average parasitaemia density of 1243.7 parasite/μL of blood. The highest (1376.9 parasite/μL of blood) and lowest (1093.2 parasite/μL) parasitaemia densities were recorded in Sista and Makwagi, respectively (Table 2). In these areas, the occurrence and intensity of malaria infection were statistically higher among farmer (71.3%, 1922.9 parasite/μL of blood) compared to non-farmer participants (*P* < 0.05). Analysis of infection for various types of farming activities revealed that rice and yam plantations were highly associated with the prevalence of infection. The highest parasite load was observed among rice farmers (2991.6 parasite/μL of blood).

### Prevalence and Intensity of Malaria Infection with Respect to Sex and Age of the Infected Participants

Both male and female of all ages were susceptible to malaria infection. Individuals within 21–30 years and ≥ 41 years of age group recorded a high rate of infections of 69.4% and 78.9%, respectively (Table 3). Generally, the rate of infection among male and female participants are comparable in this study. However, the parasitaemia load according to the age and sex of the infected participants is significantly high among children and male participants. It was found that the intensity of the infection oscillates among children and significantly reached a peak of 1896.60 parasite/μL of blood in the infected individual within the age group of 21 to 30 years (*P* = 0.034). Gender wise, infected males harboured more parasites than their female counterparts (Table 4).

### Comparative Analysis of Risk Factors Among Farmers and Non-Farmers in Study Area

Our bivariate analysis reveals that the occurrence of malaria infection was twice more likely in male farmers (90.8%, AOR: 2.428). Age groups of 6–10 years and ≥ 11 years with odd ratios of 2.168 and 3.750 were two and four times more likely to be infected with *P. falciparum* in the farmers’ population compared to children of ≤ 5 years, which had a higher prevalence of infection among non-farmers (55.0%, AOR: 2.901). The proximity of bush to human habitation influenced the occurrence of malaria infection in farmers (73.9%, AOR: 3.242) and non-farmers (38.1%, AOR: 1.362) study populations (Table 5).

## Discussion

Malaria remains the most devastating health problem in many tropical countries, particularly in Nigeria (14). The high prevalence (58.1%) and average parasite density 1243.7/μL of blood obtained in this study highlight the endemicity of infection in the study area. This outcome is similar to 58.2% and 53.3% in Ogun State and Anambra State reported by Onyido et al. (15) and Mogaji et al. (16), respectively. However, the prevalence reported by Opreh et al. (17) and Oseghale et al. (18) in Southern Nigeria were lower compared to our findings. This may be due to the variations in ecological settings, season and sampling method.

Meanwhile, the high prevalence and parasite density observed in this study could be attributed to similar environmental conditions prevailing in these localities and period of sampling, rain season that coincides with the peak of infection as previously reported by Babamale and Ugbomoiko (19). This is because the global environmental change is expected to profoundly affect the transmission of the parasites that cause human malaria. Also, the closeness of bush to most habitation provides an ideal condition for mosquitoes to thrive, thus enhancing malaria transmission in the agricultural settings.

Reports have shown that water distribution influences malaria vector populations and parasite transmission (20, 21). The significantly higher rate of infection among farmers in this study population substantiates the observation. This is similar to the reports of Onyido et al. (22) in Anambra State and Erhabor (23) in Sokoto, North-western Nigeria. This probable occupational hazard may be explained by the observations that most farmers often live very close or within their farmlands, thus exposing them to pervasive bites of vectors, which find such environment conducive for breeding. Similarly, farmers are usually fatigued at night resulting in deep sleep, which favours uninterrupted bloodsucking by mosquitoes. Indeed, farming and malaria have a long history of interconnection as reported previously (24, 25).

Our study further revealed that rice farmers recorded high occurrence of infection when compared to yam and sugar cane farmers. This observation is probably due to the fact that rice, one of the staple crops in Nigeria, relies on flooded paddies, which provide breeding sites for *Anopheles* species, a principal vector of malaria parasites. This has also been previously reported among farmers in African regions (26, 27). However, a slight deviation was observed among farmers involved in different agricultural practices in northern Tanzania (28).

The prevalence and intensity of malaria with respect to age and sex among the farmers conflict with the normal trend previously reported in endemic communities of the general population. For instance, many reports in malaria high transmission zones showed that younger age groups are often susceptible to infection (19, 29–31). However, in this current study, the age group 21–30 and ≥ 41 showed high prevalence, which is consistent with the report in Obuasi Municipality, Ghana (32). It is also noteworthy that the age group ≥ 41 had the least parasitaemia load despite having the highest prevalence recorded. A similar report was documented by Nkuo-Akenji et al. (33). This occurrence is thought to be a result of the build-up of matured immunity due to persistent exposure as reported by several authors in many endemic regions of sub-Saharan Africa (19, 34).

Overall, the data analysis of this study showed that farming activities influence the pattern and transmission of malaria infection. Unlike in non-farmer populations, infection is significantly higher among males and adult individuals that directly or indirectly engaged in farming activities. This observation is identical to the reports of Uneke et al. (35) in South-eastern Nigeria, Pathak et al. (36) and Kembhavi et al. (37) in Western India, and Muntaka et al. (38). This is because farming activities in this part of the country is actively practised by adult male participants.

Lastly, our bivariate analysis also revealed that the proximity of bush and bathroom to human habitation are essential transmission factors for malaria infection. This further suggests that the malaria vector is inextricably tied to waterlog common in the study area as previously reported (39, 40). Most of the bathrooms in our study locations were poorly built, thus stagnating water indiscriminately around human habitation. Moreover, studies have previously indicated that as little as fog, smog or water droplet on the surface of leaves in the farm is enough to support the breeding of mosquitoes (41). This may account for the higher prevalence of infection among individuals who live very close to the farmland or bush as observed in this study. This, therefore, emphasises the insistency of WHO on the need for integrated control strategies for infectious diseases, malaria inclusive. However, the inability of our research team to screen equal numbers of farmers and non-farmers as well as conducting this survey during the rainy season which coincides with the peak period of farming activities and malaria transmission may be a limitation of this study.

## Conclusion

In conclusion, farming, a sustainable means of livelihood of many rural dwellers, poses a great risk for the acquisition of malaria infection. Protection of local farmers through provision of efficient healthcare services and insecticide-treated nets should be extended to many malaria-endemic rural communities to afford them the opportunity of integrated control measures to curb the transmission of malaria infection in their various settings. Also, enlightenment on water and environmental management practices in this type of community will no doubt go a long way in reducing the incidence of the disease.

## Figures and Tables

**Table 1:** Descriptive characteristics of the study population (*n* = 341).

Variables	*n* (%)
Locations	
Shigo	87 (25.5)
Makwagi	71 (20.8)
Sista	70 (20.5)
Ogo-oluwa	113 (33.1)
Occupation	
Farmer	230 (67.4)
Non-farmer	111 (32.6)
Gender	
Male	128 (37.5)
Female	213 (62.5)
Age	
≤ 5	35 (10.3)
6–10	84 (24.6)
11+	232 (68.1)
Educational level	
Primary	55 (16.1)
Post-primary	83 (24.3)
Illiterate	203 (59.5)

Note: The classified data were subjected to Frequency Analysis stratified with selected population characteristics

**Table 2:** Prevalence and intensity of *P. falciparum* stratified by location, occupation and type of farming activities

Characteristics	Prevalence				Parasitaemia density		

	Number examined	Number infected	%	95% CI	Mean	SD	95% CI
Location							
Shigo	87	46	52.9	41.9–63.6	1101.0	732.8	883.4–1318.6
Makwagi	71	43	60.6	48.2–71.7	1093.2	753.8	861.3–1325.1
Sista	70	43	61.4	49.0–72.6	1376.9	789.5	1133.9–1619.8
Ogo oluwa	113	66	58.4	48.7–67.5	1354.5	829.0	1150.7–1558.3
*P*-value			0.688		0.132		
Occupation							
Farmers	230	164	71.3	64.9–76.9	1922.9	791.1	1078.3–2181.5
Non-farmers	111	34	30.6	22.4–40.2	1260.0	788.6	1095.8–1424.2
*P*-value			0.034		0.002		
Types of farming							
Rice farming	93	74	79.6	69.7–87.0	2991.6	840.6	824.2–3014.5
Yam farming	23	16	69.6	46.9–85.9	1256.9	179.0	879.1–1634.7
Sugar cane farming	16	6	37.5	16.3–64.1	1350.0	251.8	910.5–1432.9
Others	98	45	45.9	35.9–56.3	1243.7	767.3	1222.1–1526.0
*P*-value			0.005		0.022		
Total	341	198	58.1	52.6–63.3	1243.7	788.1	1133–1565

Note: Overall data were subjected to Chi-square analysis and one-way ANOVA analysis of means parasitaemia were tested and outcome were considered significant when with *P*-value less than 0.05. CI = confidence interval; SD = standard deviation

**Table 3:** Prevalence of falciparum malaria with respect to age and sex of the study populations

Age group	Overall			Male			Female		

	Number examined	Number infected	% (95% CI)	Number examined	Number infected	% (95% CI)	Number examined	Number infected	% (95% CI)
≤ 10	58	32	55.2 (41.6–68.0)	26	15	57.7 (37.2–76.0)	32	17	53.1 (35.0–70.5)
11–20	38	19	50.0 (34.8–65.2)	11	6	54.5 (24.6–81.8)	27	13	48.1 (29.1–67.6)
21–30	144	100	69.4 (61.1–76.7)	36	26	72.2 (54.6–85.2)	108	74	68.5 (58.8–76.9)
31–40	46	21	45.7 (31.2–60.8)	17	9	52.9 (28.5–76.1)	29	12	41.4 (24.1–6 0.9)
≥ 41	33	26	78.9 (60.6–90.4)	38	21	55.3 (38.5–71.0)	17	5	29. 4 (11.4–55.0)
*P*-value			0.006			0.534			0.005
Total	341	198	58.1 (52.6–63.3)	128	77	60.2 (51.1–68.6)	213	121	56.8 (49.9–63.5)

Notes: CI = confidence interval. Overall and age stratified data were subjected to Chi-square analysis with respect to sex and *P*-value less than 0.05 was considered for significant level

**Table 4:** Sex and age pattern of parasite density amongst infected populations

Age (years)	Overall		Male		Female	

	Mean (SD)	95% CI	Mean (SD)	95% CI	Mean (SD)	95% CI
≤ 10	1277.01 (783.57)	970.98–1393.04	1016.70 (773.64)	962.54–1193.15	844.76 (802.43)	521.67–877.21
11–20	1048.36 (811.89)	881.50–1115.22	1129.99 (908.16)	1098.75–1261.87	1015.10 (785.40)	987.56–1206.55
21–30	1896.60 (907.89)	747.05–1946.15	1976.76 (999.53)	684.11–2001.02	1269.88 (878.58)	578.03–1408.96
31–40	1644.75 (874.25)	985.13–1904.37	1648.05 (849.30)	943.99–1863.34	1342.82 (903.41)	779.05–1421.85
≥ 41	953.00 (758.28)	748.01–1057.99	1078.62 (721.36)	213.34–1039.01	895.74 (855.68)	974.90–1076.45
Total	1243.7 (788.10)	1133–1565	1184.4 (809.90)	1000.5–1368.2	1281.5 (774.90)	1141.9–1420.2
*P*-value	0.034		0.002		0.054	

Notes: Case selection one-way ANOVA analysis were tested with parasitaemia satisfied by age and sex. *P*-value less than 0.05 was considered significant. CI = confidence interval; SD = standard deviation

**Table 5:** Comparative bivariate analysis of risk factors among farmers and non-farmers in study area

Characteristics	Farmers (164)			Non-farmers (34)		

	*n*	% (95% CI)	AOR (95% CI)	*n* %	(95% CI)	AOR (95% CI)
Sex						
Male	65	90.8 (80.3–96.2)	2.428 (1.92–2.48)**	63	28.6 (18.2–41.5)	Ref
Female	165	63.6 (55.6–70.9)	Ref	48	33.3 (20.8–48.5)	1.164 (0.99–1.19)
*P*-value		0.029			0.372	
Age						
≤ 5	15	40.0 (17.5–67.1)	Ref	20	55.0 (32.0–76.2)	2.901 (2.71–3.06)**
6–10	45	86.7 (72.5–94.5)	2.168 (1.63–2.19)	39	33.3(19.6–50.3)	1.711 (1.53–1.79)
11+	170	70.0 (62.4–76.7)	3.750 (2.85–3.80)***	52	19.2 (10.1–33.0)	Ref
*P*-value		0.045			0.035	
Location						
Shigo	67	55.2 (42.6–67.2)	1.084 (0.79–1.11)	20	45.0 (23.8–68.0)	3.500 (3.35–3.75)**
Ogo-Oluwa	101	50.5 (40.4–60.5)	Ref	32	46.9 (29.5–65.0)	3.672 (2.98–3.88)**
Makwagi	40	97.5 (85.3–99.9)	1.931 (1.15–2.01)	31	12.9 (4.2–30.8)	Ref
Sisita	42	88.1 (73.6–95.5)	1.745 (1.38–1.94)	28	21.4 (9.0–41.5)	1.712 (1.62–1.74)
*P*-value		0.487			0.334	
Proximity to bush						
≤ 50 m	188	73.9 (66.9–79.9)	3.242 (2.57–3.61)***	21	38.1 (19.0–61.3)	1.362 (1.25–1.41)*
> 50 m	42	59.5 (43.3–74.0)	Ref	90	28.9 (20.1–39.5)	Ref
*P*-value		0.0213			0.698	
Bathroom						
Within the house	126	81.7 (73.7–87.8)	1.392 (1.09–1.45)	66	31.8 (21.2–44.6)	1.071 (1.01–1.21)
Outside the house	104	58.7 (48.6–68.1)	Ref	42	31.0 (18.1–47.2)	Ref
*P*-value		0.042			0.920	

Notes: The data were subjected to Chi-square statistical and one-way ANOVA analysis. *P*-value less than 0.05 was considered significant. Logistic regression analysis was done to obtain odd ratio for the comparative analysis of risk factors among farmers and non-farmers participants.

* less than 0.05;

** less than 0.001;

*** 0.001.

CI = confidence interval; AOR = adjusted odds ratio
